# Patient satisfaction and operator proficiency in gasless transaxillary endoscopic thyroidectomy under IONM: a retrospective cohort study

**DOI:** 10.3389/fendo.2024.1457571

**Published:** 2024-10-09

**Authors:** Yushuai Zhang, Yishen Zhao, Hong Tang, Hongrui Zou, Yang Li, Xuehai Bian

**Affiliations:** ^1^ Department of Thyroid Surgery, Jilin Provincial Key Laboratory of Translational Medicine in Surgery, Jilin Provincial Engineering, China-Japan Union Hospital of Jilin University, Changchun, China; ^2^ Laboratory of Thyroid Disease Prevention and Treatment, China-Japan Union Hospital of Jilin University, Changchun, China; ^3^ Department of Ultrasonography, China-Japan Union Hospital of Jilin University, Changchun, Jilin, China

**Keywords:** unilateral thyroid cancer, thyroidectomy, GTET, Qol, learning curve

## Abstract

**Background:**

This study aims to evaluate the surgical safety and effectiveness of gasless transaxillary endoscopic thyroidectomy (GTET), assess patients’ short-term perceptions and long-term outcomes, and delineate the learning curve and key surgical techniques of the operators.

**Materials and methods:**

Clinicopathological and postoperative follow-up data from patients with unilateral thyroid cancer in the same period were collected. These patients were divided into the GTET group and the traditional open surgery group to compare and analyze the differences and explore the factors affecting the learning curve of GTET.

**Results:**

Patients who chose GTET had better general health and thyroid conditions than those in the open group, and the quality of postoperative life was better in the GTET group than in the open group, with the main differences between the two groups being appearance and neck and shoulder function. The GTET learning curve in this study peaked at 19 cases, with slight differences between left and right, and a larger sample size is still needed to explore the factors affecting the learning curve.

**Conclusions:**

GTET has a reliable safety and efficacy profile for patients with unilateral thyroid cancer. Intraoperative nerve monitoring (IONM) techniques require some adaptation in GTET. In some respects, patients’ postoperative experience and quality of life are superior to those of conventional open surgery. There is a learning curve for GTET, but large samples are still needed to explore its true significance.

## 1 Introduction

With the advancement of diagnostic technologies, such as ultrasound and fine needle aspiration, thyroid cancer has become increasingly prevalent, predominantly affecting young women. Recent increases in incidence have been particularly notable in cases of papillary thyroid microcarcinoma (PTMC), characterized by a maximum tumor diameter of less than 1 cm. A report (1) on the incidence of thyroid cancer in Zhejiang Province, China shows that the sharp rise in thyroid cancer cases in the past primarily involved PTMC. Globally, thyroid cancer tends to be overtreated (2). While some studies (3–5) advocate for active surveillance or less invasive thermal ablation techniques for PTMC, these approaches remain contentious (6–8). Current guidelines in China (9) still favor more aggressive surgical interventions. Moreover, even in cases of less aggressive micropapillary thyroid cancer, Chinese patients express significant concerns about the progression of the disease and show limited confidence in active surveillance (10). Therefore, for young women, endoscopic techniques that satisfy both radical treatment principles and aesthetic considerations have been well-developed. Gasless transaxillary endoscopic thyroidectomy (GTET) has seen ongoing innovation and standardization since its introduction, earning widespread trust from physicians and preference from patients. Although previous studies (11–14) indicate a learning curve of approximately 30-60 cases for achieving proficiency in GTET, there has been limited discussion on factors influencing the duration of GTET surgery or the implications of the learning curve.

Protection of nerves and parathyroid glands remains a focal point in surgery. Injury to the recurrent laryngeal nerve (RLN) is a common complication of thyroidectomy. GTET has been associated with higher rates of temporary RLN injury (15). Unlike the RLN, the external branch of the superior laryngeal nerve (EBSLN) often receives less attention from clinicians due to its variable position and the challenges in protecting it. The EBSLN is crucial for voice quality, projection, and especially high pitches, which are vital for professionals. Intraoperative nerve monitoring (IONM) has been extensively utilized in traditional open thyroid surgeries to protect the RLN, EBSLN, and other motor nerves since its introduction. However, it has been reported that only 5% (9/160) of endoscopic or robotic thyroidectomy trials have used IONM (16). The application of endoscopy, particularly in GTET, has not been widely discussed. Our study aims to highlight the differences in IONM application in GTET and to standardize the procedure for clinical guidance.

In this study, we assessed the safety, pros, and cons of GTET, explored its learning curve, and analyzed factors affecting operative time and proficiency. We also shared insights into the challenges and benefits of neuroprotection in GTET and aimed to standardize the use of IONM.

## 2 Materials and methods

### 2.1 Study cohort

We retrospectively analyzed 185 consecutive patients with unilateral DTC, of whom 82 underwent GTET and 103 underwent conventional open thyroidectomy. All patients met the surgical indications for GTET and were informed of the differences between GTET and open surgery, with patients choosing their own surgical procedure. Specific inclusion and exclusion criteria were based on the Chinese version of the *Expert Consensus on Endoscopic Thyroidectomy by a Gasless Unilateral Axillary Approach* (version 2022) (17). Inclusion criteria included: (i) patients with differentiated thyroid cancer requiring surgical treatment; (ii) primary tumors with a maximum diameter of <4 cm; and (iii) no extra glandular invasion or tiny extravasated foci that only penetrated the anterior thyroid capsule or tiny invasion of the sternal thyroid muscle. Exclusion criteria included: (i) patients with severe comorbidities who could not tolerate general anesthesia or the conventional surgical position; (ii) DTC with obvious extra glandular invasion, such as invasion of the recurrent laryngeal nerve, larynx, trachea, esophagus, etc.; (iii) DTC with upper mediastinal lymph node metastasis or metastatic lymph node fusion and fixation; (iv) DTC with poor prognostic pathological subtypes of dedifferentiated thyroid cancer. The surgery was performed by the same experienced operator who had no prior experience with endoscopic thyroidectomy. Before performing GTET, the operator had performed over 20 thyroidectomies using the sternocleidomastoid intermuscular approach and switched to instruments from the same manufacturer as the endoscopic instruments.

### 2.2 Operation procedures

**Figure 1:**
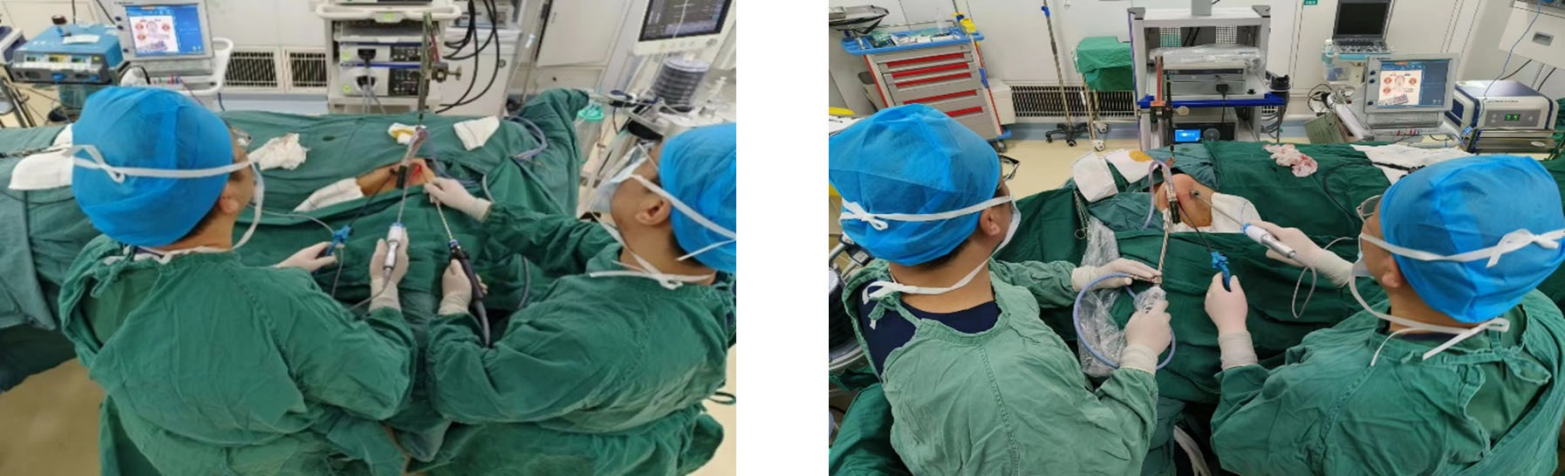
Selection of operating position. Right-handed operators performing left-sided procedures (left image) are more affected by the camera assistant compared to right-sided procedures (right image).

**Figure 2:**
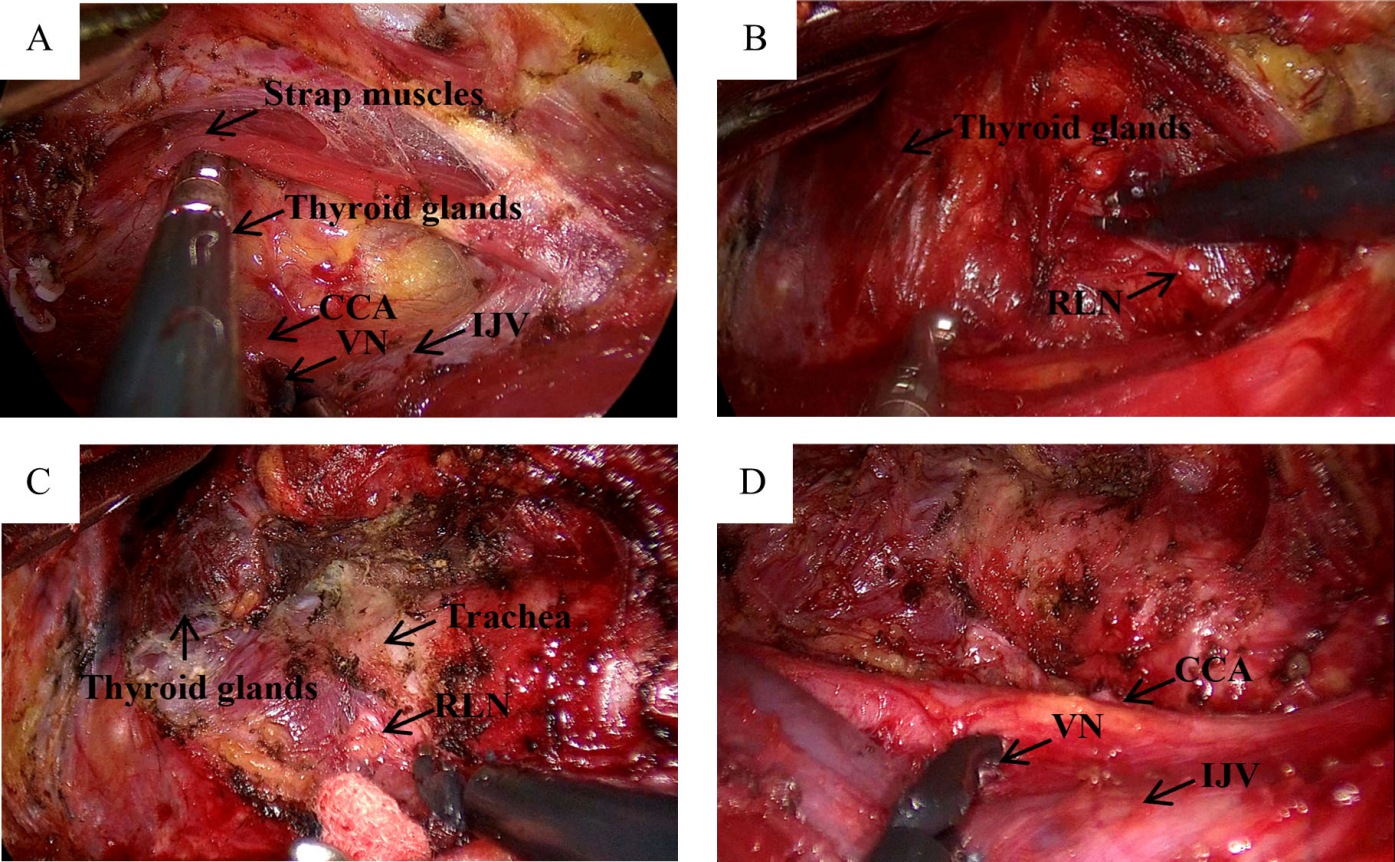
IONM monitoring of VN and RLN in GTET. **(A)** Separate the strap muscles to descend the cervical sheath and monitor the VN, recorded as V1; **(B)** “Cross” method to find RLN, monitoring signal recorded as R1; **(C)** Separate thyroid drops RLN, monitoring signal recorded as R2; **(D)** Nonitoring VN after adequate haemostasis is recorded as V2.

**Figure 3:**
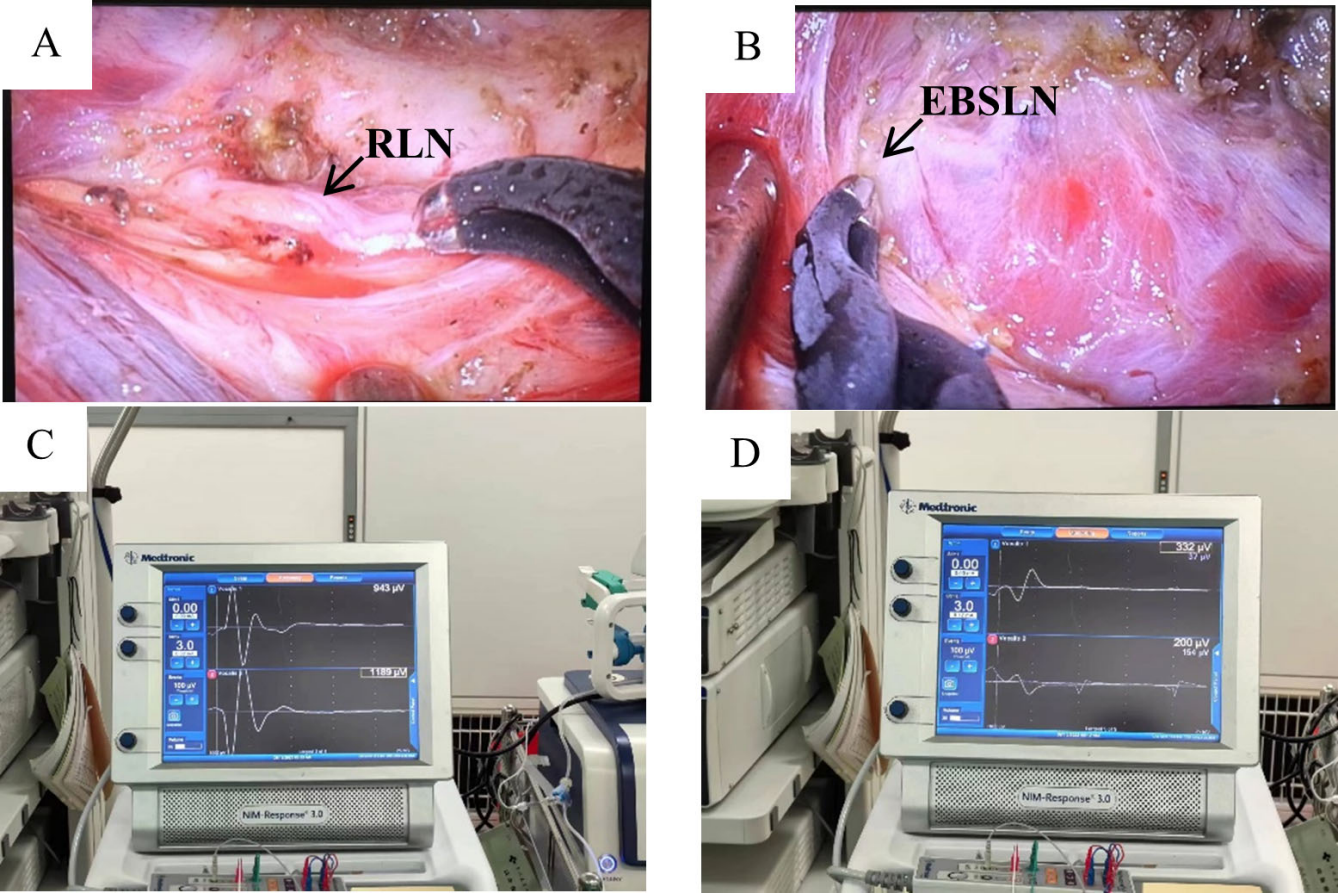
RLN and EBSLN after water rinse. **(A, C)** Postoperative RLN signal values in GTET; **(B, D)** Postoperative EBSLN signal values in GTET.

**Figure 4:**
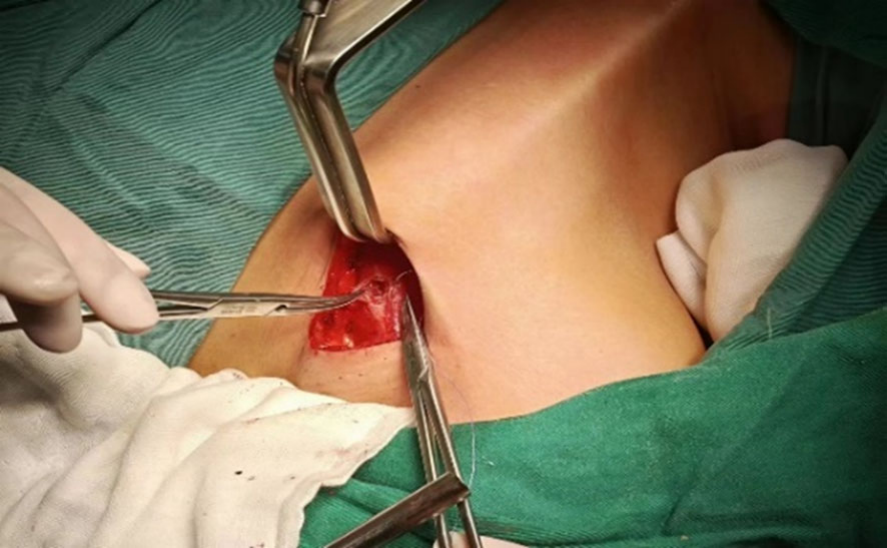
Parathyroid auto-transplantation into the pectoralis major muscle.

After resting and combined anesthesia, the patient was placed in a supine position with shoulder pads, the affected upper limb naturally abducted (60°-90°), and fixed in the armpit. The incision was made from the inner top to the outer bottom along the first or second natural skin fold line of the armpit, measuring 3.5-4.5 cm in length. Initially, with the assistance of a retractor, the flap was separated from the clavicle along the surface of the pectoralis major fascia toward the thyroid using a retractable electric knife. The hook was inserted into the upper clavicle flap, about 3.0-4.0 cm below the main incision, and a 0.5 cm incision was made at the intersection of the anterior axillary line and the outer upper margin of the breast. This smaller incision was used to insert another operating instrument through a 5 mm Trocar, as shown in **Figure 1**. On the second advancement of the hook, the natural space between the sternocleidomastoid and the head of the clavicle was identified, extending from the clavicle to the intermuscular space of the sternocleidomastoid muscle. The fat and connective tissue within this natural space were separated using an ultrasound knife, extending the upper boundary to the lower boundary of the thyroid cartilage or the upper pole of the thyroid gland, and the lower boundary to the sternal attachment point of the sternocleidomastoid muscle. On the third advancement of the hook, the separation stage progressed from the sternocleidomastoid muscle space to the thyroid area. After traversing the sternocleidomastoid muscle space, the separation continued between the medial carotid sheath (internal jugular vein) and the lateral margin of the sternothyroid muscle until the natural gap within the thyroid surgical envelope was reached. The retention of the omohyoid muscle depended on the exposure of the operative field. A suspension retractor was placed under the sternal thyroid muscle to complete the third advancement, fully exposing the thyroid gland and completing the dissociation of the upper and lower poles of the thyroid gland. If pathology confirmed malignancy, the ipsilateral central lymph nodes were treated concurrently, and the isthmus of the thyroid was severed from the front of the trachea. Intraoperative nerve protection was routinely performed using exploratory forceps, as shown in **Figure 2**. The RLN and EBSLN were re-examined at the end of the surgery, as shown in **Figure 3**. Pectoralis major muscle autografting was performed for misplaced parathyroid glands, as shown in **Figure 4**.

### 2.3 Postoperative management

Postoperative ice packs were applied to all patients upon their return to the ward nursing unit (traditional open surgery patients: neck; GTET patients: axilla to neck). Calcium supplementation was administered according to the Chinese consensus (18) and international guidelines (19). Oral levothyroxine sodium at a dosage of 50 ug/day was initiated on the second day post-surgery and continued until the first month postoperatively, at which point blood calcium and thyroid function were assessed and the dosage adjusted accordingly. The drain was removed if the ultrasound did not reveal any significant fluid accumulation in the residual thyroid cavity.

### 2.4 Postoperative follow-up

One month postoperatively, patients returned to the hospital for ultrasound, thyroid function tests, and blood calcium level checks. Patient satisfaction was assessed three months postoperatively using the University of Washington Head and Neck Cancer Questionnaire (UW-QoL), Patient and Observer Scar Assessment Scale (POSAS), Voice Handicap Index 30 (VHI-30), and the patient’s subjective feelings. The UW-QoL, a self-report questionnaire used globally to evaluate the quality of life in patients with head and neck malignancies (20), measures the patient’s quality of life. Scarring satisfaction was assessed using the patient scale portion of POSAS (21). The VHI-30 questionnaire, which evaluates voice-related quality of life, was administered to all participants under physician guidance (22).

### 2.5 Statistical analysis

Cumulative sum analysis (CUSUM) is a sequential analysis technique extensively utilized for describing surgical learning curves. The calculation formula is CUSUM _n_ =OT _n_ – OT _mean_ + Cusum_n-1_, where N represents the operation serial number arranged in chronological order, OT _n_ is the operation time of the N-th operation, OT _mean_ is the average operation time, and the curve is plotted with the operation sequence number on the horizontal axis and CUSUM on the vertical axis. A shift from a positive to a negative slope indicates the peak of the learning curve, which typically signifies the minimum number of surgeries required to surpass the learning curve threshold, thus differentiating between the learning improvement group and the proficiency group.

Data were analyzed using SPSS 27.0 statistical software. Categorical variables were expressed as frequencies and percentages (%), and analyzed using the χ2 test or Fisher’s exact test as appropriate. Normally distributed measurement data were presented as mean ± standard deviation (x ± s), and comparisons between groups were conducted using the t-test for independent samples. Non-normally distributed data were expressed as median (interquartile range), and non-parametric tests were employed for comparisons between groups. A *p*-value of <0.05 was considered statistically significant. Data visualization was carried out using Origin 2018.

## 3 Results

### 3.1 Comparison of patient characteristics

Among the 185 patients enrolled in this study, 103 underwent conventional open thyroidectomy and 82 underwent GTET. All baseline characteristics were comparable between the two groups (Group I vs Group II, see **Table 1**). Patients who chose GTET tended to be younger (45.18 ± 10.78 yrs vs 34.62 ± 7.40 yrs), women with lower BMI (25.63 ± 4.51 kg/m^2^ vs 23.25 ± 3.73 kg/m^2^), better systemic conditions (ASA Grade II: 17% vs 4.8%), and better thyroid conditions (thyroiditis: 25% vs 12%). Open surgery takes less operating time than GTET (median operating time: 65 min vs 140 min), with fewer post-operative drainage (Day 1: 41.63 ml vs 82.55 ml; Day 2: 23.70 ml vs 49.07 ml). There was no statistically significant difference in performance between GTET and open surgery in patients with different conditions (multifocal or not, difference in tumor diameter, difference in lymph node metastasis in the central group).

**Table 1:** Demographic and clinical characteristics of patients.

	Group1	Group2	p-value	Substage of surgery		p-value
	(n=103)	(n=82)		Learning stage	Proficiency stage	
**Age(yrs)**	45.18 ± 10.78	34.62 ± 7.40	<0.001	35.79 ± 8.92	34.27 ± 6.92	0.436
**Sex**			0.139			0.278
Female	79	70		18	52	
Male	24	12		1	11	
**BMI (kg/m^2^)**	25.63 ± 4.51	23.25 ± 3.73	<0.001	22.51 ± 2.65	23.48 ± 3.99	0.324
**ASA grading**			0.011			0.930
Grade I	85	78		18	60	
Grade II	18	4		1	3	
**TI-RADS classification**			0.554			
4	47	41		11	30	0.432
5	56	41		8	33	
**Location**						
Left	54	38	0.411	8	30	0.673
Right	49	44		11	33	
Top	27	22	0.785	4	18	0.107
Middle	45	39		13	26	
Bottom	31	21		2	19	
**BRAF mutation**			0.811			0.281
Positive	73	61		17	44	
Negative	13	8		1	7	
Unknown	17	13		1	12	

BMI, body mass index; ASA, American Society of Anesthesiologists; TI-RADS, Thyroid imaging reporting and data system; BRAF refer in particular to BRAF gene V600E.

### 3.2 Comparison of IONM techniques

Nerve identification and monitoring were performed using a traditional probe in open surgery (Group I) and endoscopic stimulating dissecting forceps in endoscopic surgery (Group II). Intraoperative vagal and recurrent laryngeal nerve monitoring values are shown in **Table 2**. Significant reductions in vagal and recurrent laryngeal nerve signals occurred intraoperatively in five individuals in both Group I and Group II (Learning stage: 1; Proficiency stage: 4). All were type I injuries, and none of the patients developed loss of signal (LOS). Ten patients developed varying degrees of hoarseness postoperatively. No vocal cord paralysis was observed by laryngoscopy at 3 months postoperatively in any of the patients (see **Table 2**).

**Table 2:** Comparison of neuromonitoring scales.

IONM signal	EMG amplitude (μV)				P value
	Group I	P value	Group II	P value	
V		0.136		0.972	
V1	1230 ± 203		1317 ± 181		0.544
V2	1350 ± 274		1319 ± 378		0.516
R		0.374		0.752	
R1	1309 ± 218		1309 ± 181		0.990
R2	1277 ± 294		1295 ± 345		0.700

EMG, electromyographic.

### 3.3 Comparison of postoperative symptoms and quality of life

**Figure 5:**
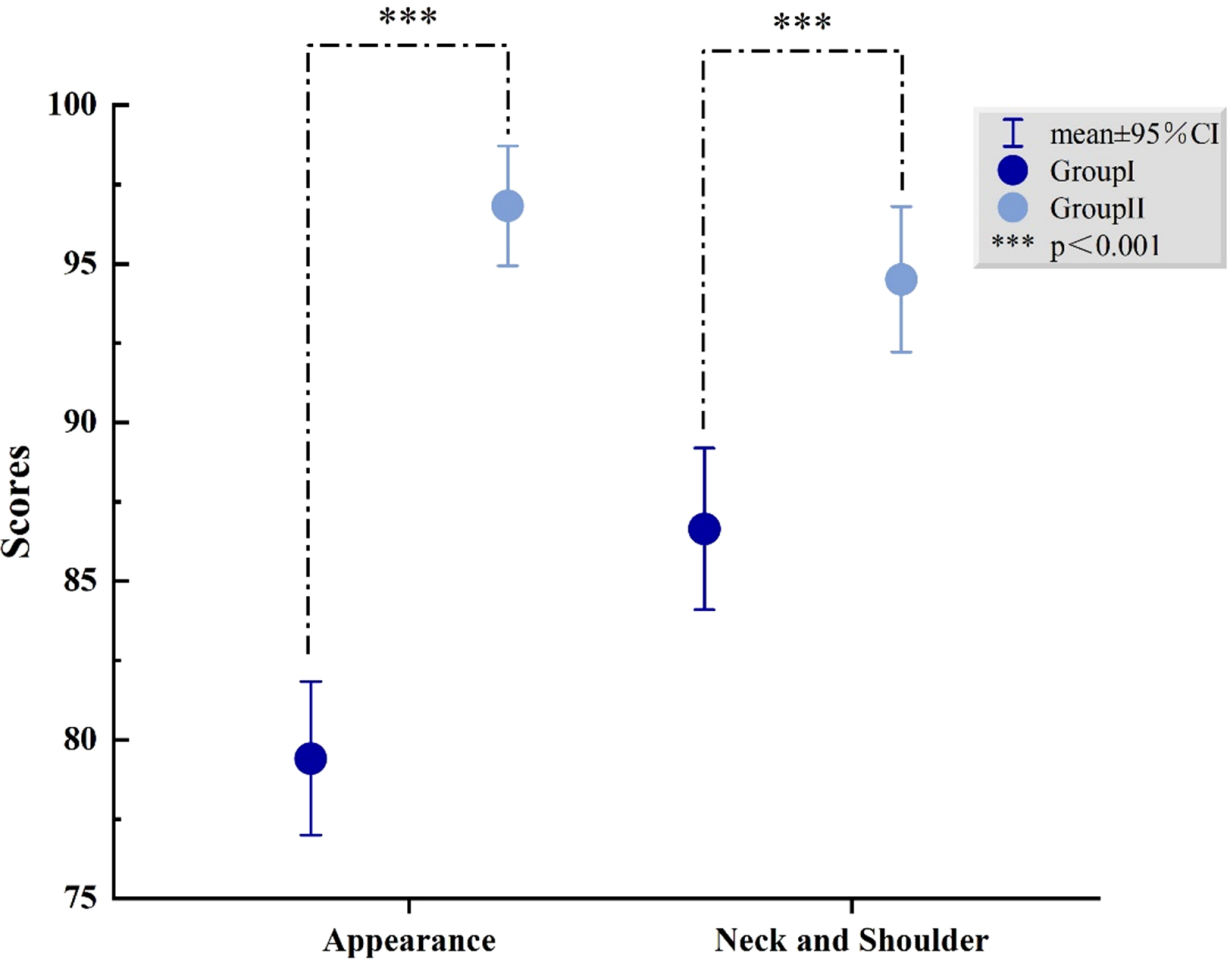
Comparison of postoperative quality of life.

To explore postoperative patient satisfaction and follow-up, comparisons were made between the conventional open thyroidectomy group (Group I) and the GTET group (Group II), as well as between the different stages of GTET (learning group and proficient group). We found that patients in the GTET group were more satisfied with their appearance results (79.42 ± 12.35 vs 96.83 ± 8.59, *P*<0.001) and neck and shoulder function (87.38 ± 13.04 vs 94.51 ± 10.41, *P*<0.001) at three months postoperatively. Additionally, there were no statistical differences between Group I and Group II in pain (86.21 ± 9.30 vs 84.63 ± 8.49, *P*=0.230), activity (87.96 ± 13.24 vs 89.27 ± 13.77, *P*=0.513), recreation (92.82 ± 10.42 vs 94.88 ± 9.84, *P*=0.170), swallowing (96.46 ± 8.65 vs 96.95 ± 8.23, *P*=0.693), chewing (99.68 ± 3.28 vs 99.19 ± 5.17, *P*=0.435), speech (94.42 ± 11.03 vs 95.73 ± 9.47, *P*=0.393), tasting (84.71 ± 14.53 vs 85.37 ± 16.64, *P*=0.775), saliva (96.84 ± 10.89 vs 98.78 ± 5.42, *P*=0.117), mood (86.80 ± 14.97 vs 88.54 ± 16.34, *P*=0.452), and anxiety (86.17 ± 14.74 vs 88.72 ± 14.78, *P*=0.244). When asked about the biggest problems after thyroid surgery, about 43.7% (45/103) of the patients in the open group complained about scarring from the neck incision and neck discomfort. In the open group, 23.3% (24/103) of patients reported changes in their voice, such as hoarseness or a lowered pitch, compared to the preoperative period. In contrast, patients in the GTET group reported fewer concerns regarding scarring. Specifically, only 20.7% (17/82) of patients in the GTET group noted changes in their voice. Likewise, 20.7% (17/82) of these patients experienced varying degrees of discomfort, ranging from numbness to pain, in the skin surrounding the incision area, which includes the shoulder, chest, and collarbone. There was no statistically significant difference between the two surgical stages in the GTET group at any follow-up, as shown in **Table 3**. The difference in cosmetic satisfaction and neck and shoulder function between the two groups is shown in **Figure 5**.

**Table 3:** Comparison of postoperative quality of life.

	Group I	Group II	p-value	Substage of surgery		p-value
				Learning group	Proficient group	
**POSAS** (scores)	46.39 ± 23.57	42.24 ± 22.26	0.225	39.37 ± 20.51	42.89 ± 22.81	0.548
**VHI** (scores)	76.50 ± 33.44	72.65 ± 33.76	0.532	62.74 ± 19.87	76.14 ± 36.29	0.128
**UW-Qol** (scores)	89.28 ± 5.16	93.13 ± 4.57	<0.001	92.60 ± 5.90	93.10 ± 4.37	0.684
**Ca** (mmol/l)	2.39 ± 0.15	2.38 ± 0.11	0.675	2.36 ± 0.08	2.39 ± 0.12	0.219
**TSH** (mIU/l)	1.92 ± 1.44	1.54 ± 1.15	0.052	1.40 ± 1.09	1.58 ± 1.17	0.541

### 3.4 Learning curve for CUSUM calculations

**Figure 6:**
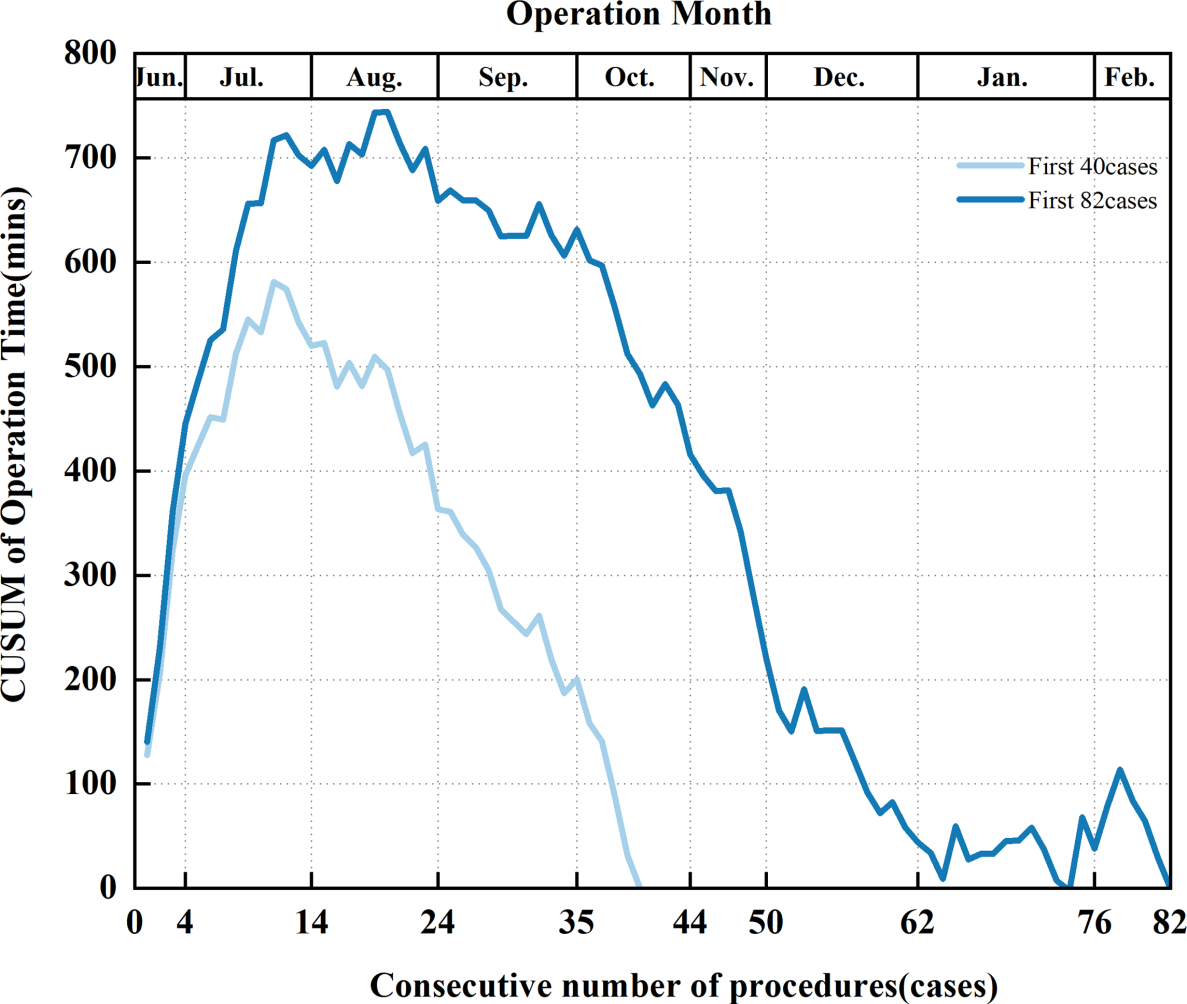
CUSUM learning curves for the first 82 and first 40 GTET cases.

**Figure 7:**
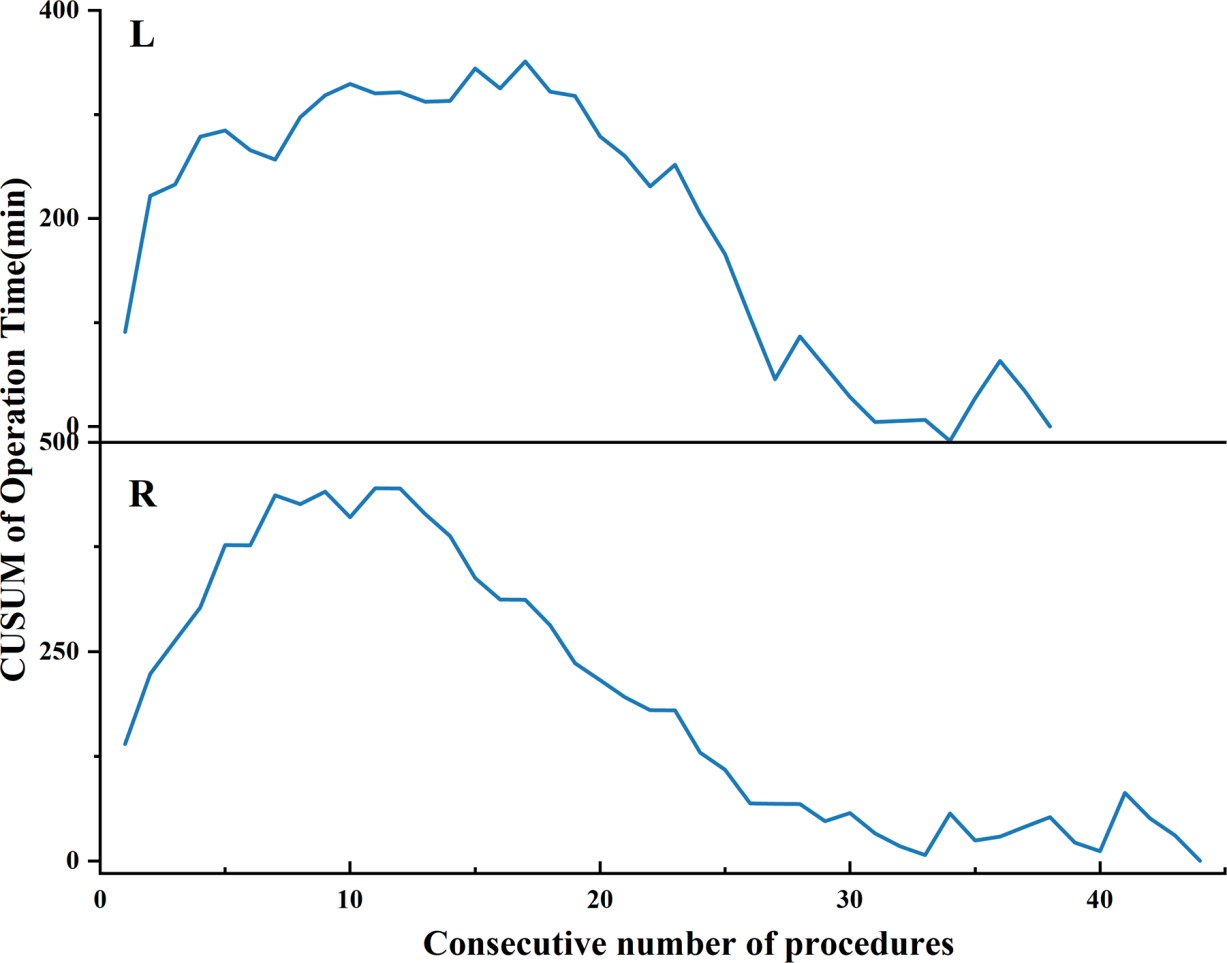
CUSUM Learning Curve for Left and Right GTET L, Lef; R, Right.

CUSUM analysis was performed in 40 and 82 cases respectively, as shown in **Figure 6**. We found that with the increasing number of cases and the decreasing median operation time (from 190 min to 120 min), the peak of the learning curve changed (from 10 to 19 cases). These 19 examples are divided into two stages: Learning stage and Proficiency stage. All baseline characteristics were comparable between the two groups (Learning stage vs Proficiency stage, see **Table 1**). The learning curves were plotted separately for the left and right sides using CUSUM analysis. We observed that the learning curve on the left side lasted longer at the peaks, as shown in **Figure 7**. The basic information about the patients in the left and right groups is shown in **Table 4**.

**Table 4:** Demographic and clinical characteristics of GTET.

	R(n=44)	L(n=38)	p-value
**Age**(yrs)	34.52 ± 7.36	34.74 ± 7.55	0.897
**Sex**			0.367
Female	36	34	
Male	8	4	
**BMI** (kg/m^2^)	23.47 ± 3.78	23.00 ± 3.71	0.575
**Location**			
Top	15	7	
Middle	21	18	
Bottom	8	13	
**Operating time**(min)	140(121,162) *	142.5(120,171.25) *	0.974
**Multifocal tumor**			0.548
Yes	7	8	
No	37	30	
**Largest tumor diameter**(cm)	0.5(0.4,0.6) *	0.5(0.28,0.8) *	0.862
**Thyroiditis**			0.804
Yes	5	5	
No	39	33	
**Metastasized central lymph node**	0(0,1) *	0(0,1) *	0.839
**Removed central lymph node**	3.5(1.25,7) *	4(2,5) *	0.718

L, Left thyroid disease; R, Right thyroid disease; T, Total patients.

* median (lower quartile, upper quartile).

## 4 Discussion

There is a recognized issue of overtreatment of the thyroid currently, yet most Chinese patients continue to prefer surgical intervention due to fears of malignancy and a limited understanding of active surveillance (10). Previous studies (23, 24) have demonstrated that GTET offers superior cosmetic outcomes and quality of life compared to open surgery, particularly regarding throat/mouth complications one month post-surgery, primarily due to damage to the anterior cervical muscle in open procedures. We analyzed the differences between GTET and traditional open surgery. From an objective standpoint, the perioperative biochemical and electrophysiological parameters, as well as the long-term prognosis of patients undergoing GTET, were found to be on par with those of conventional open surgery, affirming GTET as a safe and effective surgical option that is both reliable and preferable. Subjectively, GTET provided better cosmetic satisfaction compared to conventional open surgery, despite the incisions being significantly larger than those of other endoscopic approaches. Notably, some patients opting for endoscopic thyroidectomy developed keloid scars. Additionally, patients in the GTET group exhibited increased postoperative drainage, yet no significant dark fluid areas were observed on ultrasound on the second postoperative day, indicating that the fluid was predominantly tissue exudate.

Some scholars have raised concerns regarding the efficacy of GTET in removing lymph nodes from region VI. According to one study (14), there is no apparent learning curve for the quantity of central lymph nodes retrieved during GTET, suggesting that the number of lymph nodes removed does not vary with the surgeon’s skill level. Our research also found no significant differences between GTET and open surgery in terms of clearance of central group lymph nodes. The surgical approach of GTET involves layer-by-layer tissue isolation and descent from the lateral posterior aspect up to the thyroid gland, which poses a considerable adaptation challenge for surgeons accustomed to open procedures. Nevertheless, the protection of nerves and parathyroid glands remains a significant surgical hurdle. Protecting the parathyroid glands during GTET proves more challenging due to occasional compromised blood supply to the inferior parathyroid glands during central lymph node dissection. Thus, parathyroid autotransplantation becomes critically important in these surgeries. It has been documented that parathyroid autotransplantation in endoscopic thyroidectomy for radical thyroid cancer enhances the thoroughness of central group lymph node dissection and significantly reduces the risk of permanent hypoparathyroidism (25, 26). Another study (27) on endoscopic thyroidectomy recommends that the number of grafts should not exceed two. In terms of autotransplantation sites, our center has opted for the pectoralis major muscle, while other reports suggest using the brachioradialis muscle of the non-dominant forearm (25–27). Previous research (28) indicates that preserving at least one parathyroid gland with an intact blood supply can prevent permanent hypoparathyroidism in cases where autotransplantation is not performed. Therefore, GTET combined with parathyroid autotransplantation has shown favorable outcomes for managing hypocalcemia and hypoparathyroidism post-unilateral thyroid cancer surgery in our studies. YUAN et al. have also demonstrated that GTET outperforms other surgical techniques in managing transient hypoparathyroidism but shows poorer results for transient recurrent laryngeal nerve injury (29).

GTET enables the surgeon to operate from the lateral and posterior aspects of the thyroid gland, enhancing the visibility of critical nerves such as the VN, RLN, and EBSLN. As a result, some surgeons might neglect the use of IONM. Previous studies on patients undergoing endoscopic or robotic thyroidectomy report that only 30% monitored the VN, 25% monitored the EBSLN, and 75% routinely used IONM in all endoscopic and robotic procedures (16). It has been noted that GTET has a higher rate of temporary nerve injury compared to other endoscopic techniques (15). Various studies (23, 24, 30–33) have documented temporary RLN injury rates ranging from 1.8%-8.5% and permanent injury rates from 0%-2.4% following GTET, including two studies using modified approaches (32, 33). While the use of IONM in open surgery is well-established, GTET requires specific adjustments and enhancements to ensure its effectiveness, practicality, and customization. In this study, we share our experiences and challenges using exploratory forceps for neuromonitoring in GTET.

Exposure of the recurrent laryngeal nerve can be achieved using the bridge and tunnel approach. Increasing the stimulation current may increase the likelihood of false negatives due to fluid conduction, i.e., no nerve damage is detected by IONM, but actual nerve damage is present. Keeping the field of view clean and isolating the nerves with gauze strips intraoperatively helps to reduce both the incidence of false negatives and potential nerve damage due to heat conduction during energy instrumentation. As the upstream nerve of the recurrent laryngeal nerve, a good vagal signal can both indicate the effectiveness of the IONM system and verify the functional status of the recurrent laryngeal nerve (34). In addition, 96% of the VN is located on the deep surface of the carotid sheath and 73% of the VN is situated between the arteries and veins on the deep surface (34). Due to the need to separate and sink the carotid sheath in GTET, it is difficult to detect the signal from the VN with the exploratory forceps (or by increasing the current to 5.0 mA), and it is not possible to detect the signal from the contralateral VN. Although percutaneous probes can reach the bilateral VN in appropriate locations, safety cannot be guaranteed due to the lack of direct vision. Therefore, the use of IONM in GTET needs to pay more attention to the exclusion of false-positive (35, 36) (anesthesia-related, stimulation end-related, recording end-related, and line connection-related issues) and false-negative events (increasing the current may give a normal signal in a type I injury (36), i.e., segmental or localized RLN injury, due to conduction).

IONM plays an increasingly significant role in GTET. The protection of the RLN remains a primary concern during thyroid surgery, but the importance of protecting the EBSLN is often underestimated by surgeons due to its mild and atypical symptoms following injury (37). A study (38) examining surgeons’ attitudes toward the EBSLN revealed that only about half of the surgeons routinely identified this nerve. International guidelines (39) advocate for the use of IONM throughout the dissection of the upper pole of the thyroid gland. Similarly, the Chinese consensus (40) recommends routine monitoring of the EBSLN during endoscopic or robotic thyroidectomy. The primary causes of EBSLN injury during endoscopic thyroidectomy are thermal and traction injuries, largely because this technique relies heavily on energy devices for coagulating blood vessels (16, 41). Utilizing IONM significantly enhances the identification of the EBSLN, particularly at high magnification and/or with a 30° endoscope field of view, and helps minimize EBSLN damage (42). Protective strategies, such as the “positioning method” and “evading method,” are used during endoscopic procedures to protect the EBSLN when addressing the superior aspects of the thyroid gland. Moreover, manipulations in the supraclavicular fossa and the abduction of the patient’s arm during the axillary approach can cause damage to the brachial plexus nerves. A study (43) involving robotic axillary surgery with IONM, including somatosensory evoked potentials and motor nerve evoked potentials, has shown promise in the early identification of injuries to the brachial plexus nerves. Therefore, it is advisable for beginners to pay particular attention to the effective use of IONM.

Previous studies have indicated that mastering GTET may require about 30-60 cases. In our study, which included 19 cases, the learning curve peaked at 19 cases. This may be attributed to several factors: (1) We performed some lateral approach surgeries, which helped to familiarize the surgical team with the anatomical pathway. (2) We transitioned from using conventional open surgery instruments to those used in endoscopic approaches, such as ultrasonic scalpels and retractable electroknives, facilitating better adaptation to endoscopic procedures. (3) Both the surgeon and the assistant, who holds the mirror, were trained by the same professionals, focusing not only on the specific surgical steps but also on improving coordination and the precise movements of experienced professionals. CUSUM analysis is the most commonly utilized method for assessing surgical learning curves. In our research, we collected data from 74 GTET procedures to analyze the learning curve using CUSUM. All surgeries were performed by the same surgeon. We attempted to differentiate between the left and right sides to explore how bilateral operations influence surgeons, and we plotted the learning curves for various cases to investigate the potential limitations of using CUSUM.

In terms of spatial considerations, it is important to note that operations on the left and right sides exhibit slight differences. The camera holder, positioned at the patient’s head, creates more interference between the lens and critical instruments such as the ultrasonic scalpel when a right-handed surgeon operates on the left side. Although there was no variability in the duration of operations on the left and right sides, there was a notably longer plateau in the learning curve on the left side. In terms of time, we observed that as the total number of surgeries increased from 40 to 74, the peak of the CUSUM curve shifted from 11 to 19 when the median operative time decreased from 190 minutes to 120 minutes. This suggests that with a reduction in average operation time and an increase in the number of operations, the peak of the CUSUM curve tends to shift rightward. A study (44) analyzing thousands of cases across three different procedures (prostatectomy, malignant hysterectomy, and inguinal hernia) indicates that the peak of the CUSUM curve consistently rises with an increase in the number of procedures (from 50 to 100). Furthermore, the learning curve outlined by Jasaitis et al. peaked twice, which suggests a subjective selection of some patients with lower BMI, more suitable for endoscopic thyroidectomy, potentially due to differences in patient BMI before and after (12). Therefore, we identified some issues with the CUSUM analysis method, which seems not to accurately reflect the actual dynamics of the learning curve.

It is well known that the focus and difficulty of GTET lie in working space making. One study (13) has divided the whole operation into working space making and ipsilateral thyroidectomy, and the results show that the number of cases with a peak learning curve of the former (45 cases) is much higher than the latter (25 cases) and is closer to the total endoscopic GTET approach.

Our study has some limitations: on the one hand, the sample size is small and from the same source, which may lead to subjective bias. On the other hand, all patients in our study had unilateral thyroid cancer with mild disease, so we chose a simpler follow-up with a shorter duration, which may have overlooked some of the advantages and disadvantages of GTET. It should also be noted that we measured the operation time from the beginning of the incision to the closure of the incision and did not discuss the different stages of the operation separately, which may make some factors less influential.

## 5 Conclusions

GTET with IONM has a reliable safety and efficacy profile for patients with unilateral thyroid cancer. In some respects, patients’ postoperative experience and quality of life are superior to those of conventional open surgery. There is a learning curve for GTET, but larger samples are still needed to explore its true significance.

## Data Availability

The original contributions presented in the study are included in the article/**Supplementary Material**. Further inquiries can be directed to the corresponding author.

## References

[1] Du L Wang Y Sun X Li H Geng X Ge M. Thyroid cancer: trends in incidence, mortality and clinical-pathological patterns in Zhejiang Province, Southeast China. BMC Cancer. (2018) 18:291. doi: 10.1186/s12885-018-4081-7 29544469 PMC5856225

[2] Krajewska J Kukulska A Oczko-Wojciechowska M Kotecka-Blicharz A Drosik-Rutowicz K Haras-Gil M. Early diagnosis of low-risk papillary thyroid cancer results rather in overtreatment than a better survival. Front Endocrinol. (2020) 11. doi: 10.3389/fendo.2020.571421 PMC7573306 33123090

[3] Carlisle KM Brown JP Kim J Turner DJ Slejko JF Kuo JH. Age-stratified comparison of active surveillance versus radiofrequency ablation for papillary thyroid microcarcinoma using decision analysis. Surgery. (2024) 175:153–60. doi: 10.1016/j.surg.2023.06.054 PMC10845124 37872047

[4] Koot A Soares P Robenshtok E Locati LD de la Fouchardiere C Luster M. Position paper from the Endocrine Task Force of the European Organisation for Research and Treatment of Cancer (EORTC) on the management and shared decision making in patients with low-risk micro papillary thyroid carcinoma. Eur J Cancer. (2023) 179:98–112. doi: 10.1016/j.ejca.2022.11.005 36521335

[5] Sugitani I Ito Y Takeuchi D Nakayama H Masaki C Shindo H. Indications and strategy for active surveillance of adult low-risk papillary thyroid microcarcinoma: consensus statements from the Japan association of endocrine surgery task force on management for papillary thyroid microcarcinoma. Thyroid. (2021) 31:183–92. doi: 10.1089/thy.2020.0330 PMC7891203 33023426

[6] Cernea CR Matos LL Eugênio C Ferreira GM Cerqueira YS Leite AKN. Active surveillance of thyroid microcarcinomas: a critical view. Curr Oncol Rep. (2022) 24:69–76. doi: 10.1007/s11912-021-01177-w 35061193

[7] Maniam P Harding N Li L Adamson R Hay A Nixon I. Active surveillance for PTMC warranted for the UK population? Clin Otolaryngol. (2022) 48:88–93. doi: 10.1111/coa.13987 36183341

[8] Orloff LA Noel JE Stack BC Russell MD Angelos P Baek JH. Radiofrequency ablation and related ultrasound-guided ablation technologies for treatment of benign and Malignant thyroid disease: An international multidisciplinary consensus statement of the American Head and Neck Society Endocrine Surgery Section with the Asia Pacific Society of Thyroid Surgery, Associazione Medici Endocrinologi, British Association of Endocrine and Thyroid Surgeons, European Thyroid Association, Italian Society of Endocrine Surgery Units, Korean Society of Thyroid Radiology, Latin American Thyroid Society, and Thyroid Nodules Therapies Association. Head Neck. (2021) 44:633–60. doi: 10.1002/hed.26960 34939714

[9] Chinese Society of Endocrinology Chinese Society of Surgery Thyroid and Metabolic Surgery Group Head and Neck Tumour Committee of the Chinese Anti-Cancer Association. Guidelines for the diagnosis and treatment of thyroid nodules and differentiated thyroid cancer (second edition). Chin J Endocrinol Metab. (2023) 39:181–226. doi: 10.3760/cma.j.cn311282-20221023-00589-1

[10] Zhu P Zhang Q Wu Q Shi G Wang W Xu H. Barriers and facilitators to the choice of active surveillance for low-risk papillary thyroid cancer in China: A qualitative study examining patient perspectives. Thyroid. (2023) 33:826–34. doi: 10.1089/thy.2022.0347 36719782

[11] Chen W Yu S Sun B Wu C Li T Dong S. The learning curve for gasless transaxillary posterior endoscopic thyroidectomy for thyroid cancer: a cumulative sum analysis. Updates Surg. (2023) 75:987–94. doi: 10.1007/s13304-023-01492-w 36976499

[12] Jasaitis K Skimelyte M Maleckas A Dauksiene D Krasauskas V Gulbinas A. Transaxillary gasless endoscopic hemithyroidectomy versus conventional open hemithyroidectomy: early single-centre experience. Updates Surg. (2022) 74:917–25. doi: 10.1007/s13304-022-01286-6 35489003

[13] Sun B Li P Cong R Zhou D Zhang Z Xia F. Gasless endoscopic transaxillary thyroid surgery: CUSUM analysis of a single surgeon’s experience from 105 preliminary procedures. Surg Endoscopy. (2022) 36:8270–9. doi: 10.1007/s00464-022-09273-z 35680669

[14] Kwak HY Kim SH Chae BJ Song BJ Jung SS Bae JS. Learning curve for gasless endoscopic thyroidectomy using the trans-axillary approach: CUSUM analysis of a single surgeon’s experience. Int J Surg. (2014) 12:1273–7. doi: 10.1016/j.ijsu.2014.10.028 25448644

[15] de Vries LH Aykan D Lodewijk L Damen JAA Borel Rinkes IHM Vriens MR. Outcomes of minimally invasive thyroid surgery – A systematic review and meta-analysis. Front Endocrinol. (2021) 12. doi: 10.3389/fendo.2021.719397 PMC8387875 34456874

[16] Dionigi G Kim HY Wu CW Lavazza M Materazzi G Lombardi CP. Neuromonitoring in endoscopic and robotic thyroidectomy. Updates Surg. (2017) 69:171–9. doi: 10.1007/s13304-017-0442-z 28439772

[17] Thyroid Cancer Specialised Committee of the Chinese Anti-Cancer Association Thyroid Tumour Specialised Committee of the Chinese Society of Oncology Thyroid Disease Specialised Committee of the Chinese Society of Research Hospitals Lumpectomy/Intelligent Robot Surgery Group of the Thyroid Cancer Specialised Committee of the Chinese Anti-Cancer Association. Expert consensus on lumpectomy for thyroid surgery with inflatable axillary approach (2022 edition). Chin J Endocrine Surg. (2021) 2021:557–63. doi: 10.3760/cma.j.cn.115807-20211116-00349

[18] Sun H Tian W Jiang K Chiang F Wang P Huang T. Clinical guidelines on intraoperative neuromonitoring during thyroid and parathyroid surgery. Ann Trans Med. (2015) 3:213. doi: 10.3978/j.issn.2305-5839.2015.08.21 PMC4583592 26488009

[19] Orloff LA Wiseman SM Bernet VJ Fahey TJ 3rd Shaha AR Shindo ML. American thyroid association statement on postoperative hypoparathyroidism: diagnosis, prevention, and management in adults. Thyroid. (2018) 28:830–41. doi: 10.1089/thy.2017.0309 29848235

[20] Kim BH Ryu SR Lee JW Song CM Ji YB Cho SH. Longitudinal changes in quality of life before and after thyroidectomy in patients with differentiated thyroid cancer. J Clin Endocrinol Metab. (2024) 109:1505–16. doi: 10.1210/clinem/dgad748 38141213

[21] Thiagarajan S Menon A Panmand H Bamane P Pawar A. A prospective study to assess cervical scar satisfaction following conventional open thyroidectomy for thyroid cancer. Eur Arch oto-rhino-laryngology: Off J Eur Fed Oto-Rhino-Laryngological Societies (EUFOS): affiliated German Soc Oto-Rhino-Laryngology - Head Neck Surgery. (2024) 281:4363–72. doi: 10.1007/s00405-024-08668-z 38676715

[22] Veldova Z Holy R Rotnagl J Younus T Hlozek J Astl J. Influence of recurrent laryngeal nerve transient unilateral palsy on objective voice parameters and on voice handicap index after total thyroidectomy (Including thyroid carcinoma). Int J Environ Res Public Health. (2021) 18:4300. doi: 10.3390/ijerph18084300 33919592 PMC8072641

[23] Li T Zhang Z Chen W Yu S Sun B Deng X. Comparison of quality of life and cosmetic result between open and transaxillary endoscopic thyroid lobectomy for papillary thyroid microcarcinoma survivors: A single-center prospective cohort study. Cancer Med. (2022) 11:4146–56. doi: 10.1002/cam4.v11.22 PMC9678099 35470574

[24] Xu T Qin X Zhang Y Li P Ran Y Fan Y. A prospective study comparing the gasless endoscopic thyroidectomy trans-axillary approach to conventional open thyroidectomy: health and quality of life outcomes. Surg Endosc. (2024) 38 (4):1995–2009. doi: 10.1007/s00464-024-10689-y 38396084

[25] Cheng X Li Y Chen L. Efficacy of parathyroid autotransplantation in endoscopic total thyroidectomy with CLND. Front Endocrinol (Lausanne). (2023) 14:1193851. doi: 10.3389/fendo.2023.1193851 37441504 PMC10334188

[26] Zhang Q Qu KP Wang ZS Gao JW Zhang YP Cao WJ. Clinical application of parathyroid autotransplantation in endoscopic radical resection of thyroid carcinoma. Front Oncol. (2022) 12:942488. doi: 10.3389/fonc.2022.942488 35992841 PMC9386417

[27] Wang Z Zhang Q Gao J Cao T Zhang Y Qu K. Investigating the optimal parathyroid autotransplantation strategy in transareolar endoscopic thyroidectomy: A retrospective cohort study. Asian J Surg. (2024) 47:886–92. doi: 10.1016/j.asjsur.2023.10.036 37879989

[28] Song CM Jung JH Ji YB Min HJ Ahn YH Tae K. Relationship between hypoparathyroidism and the number of parathyroid glands preserved during thyroidectomy. World J Surg Oncol. (2014) 12:200. doi: 10.1186/1477-7819-12-200 25000948 PMC4105163

[29] Yuan Y Pan B Tang E Mo H Zhu J Yi Z. Surgical methods of total thyroidectomy for differentiated thyroid cancer: a systematic review and Bayesian network meta-analysis. Int J Surg. (2024) 110:529–40. doi: 10.1097/JS9.0000000000000819 PMC10793844 37916941

[30] Kim EY Lee KH Park YL Park CH Lee CR Jeong JJ. Single-incision, gasless, endoscopic trans-axillary total thyroidectomy: A feasible and oncologic safe surgery in patients with papillary thyroid carcinoma. J Laparoendosc Adv Surg Tech A. (2017) 27:1158–64. doi: 10.1089/lap.2016.0669 28402746

[31] Lang BH Wong KP. A comparison of surgical morbidity and scar appearance between gasless, transaxillary endoscopic thyroidectomy (GTET) and minimally invasive video-assisted thyroidectomy (VAT). Ann Surg Oncol. (2013) 20:646–52. doi: 10.1245/s10434-012-2613-y PMC3560957 22941166

[32] Lee M-C Park H Choi IJ Lee B-C Lee G-H. Comparative study of a gasless transaxillary approach versus a bilateral axillo-breast approach for endoscopic thyroidectomy in a single institute. Head Neck. (2014) 36:702–8. doi: 10.1002/hed.23349 23606356

[33] Lee J Lee JH Nah KY Soh EY Chung WY. Comparison of endoscopic and robotic thyroidectomy. Ann Surg Oncol. (2011) 18:1439–46. doi: 10.1245/s10434-010-1486-1 21184192

[34] Dionigi G Chiang FY Rausei S Wu CW Boni L Lee KW. Surgical anatomy and neurophysiology of the vagus nerve (VN) for standardised intraoperative neuromonitoring (IONM) of the inferior laryngeal nerve (ILN) during thyroidectomy. Langenbecks Arch Surg. (2010) 395:893–9. doi: 10.1007/s00423-010-0693-3 20652584

[35] Zhang D Wu CW Wang T Zhao Y Kim HY Pino A. Drawbacks of neural monitoring troubleshooting algorithms in transoral endoscopic thyroidectomy. Langenbecks Arch Surg. (2021) 406:2433–40. doi: 10.1007/s00423-021-02217-6 34264393

[36] Sun H Tian W Chinese Thyroid Association CoSCMDA Chinese Research Hospital Association Thyroid Disease C. Chinese guidelines on intraoperative neuromonitoring in thyroid and parathyroid surgery (2023 edition). Gland Surg. (2023) 12:1031–49. doi: 10.21037/gs-23-284 PMC10493630 37701297

[37] Delbridge L. The ‘neglected’ nerve in thyroid surgery: the case for routine identification of the external laryngeal nerve. ANZ J Surg. (2001) 71:199. doi: 10.1046/j.1440-1622.2001.02120.x 11355722

[38] Almquist M Nordenstrom E. Management of the exterior branch of the superior laryngeal nerve among thyroid surgeons - Results from a nationwide survey. Int J Surg. (2015) 20:46–51. doi: 10.1016/j.ijsu.2015.06.022 26074288

[39] Barczynski M Randolph GW Cernea CR Dralle H Dionigi G Alesina PF. External branch of the superior laryngeal nerve monitoring during thyroid and parathyroid surgery: International Neural Monitoring Study Group standards guideline statement. Laryngoscope. (2013) 123 Suppl 4:S1–14. doi: 10.1002/lary.v123.S4 23832799

[40] Sun H Tian W. Expert consensus on protection and monitoring of the external branch of the superior laryngeal nerve during thyroid and parathyroid surgery (2017 edition). Chin J Pract Surg. (2017) 37:1243–9. doi: 10.19538/j.cjps.issn1005-2208.2017.11.14

[41] Dionigi G Boni L Rovera F Bacuzzi A Dionigi R. Neuromonitoring and video-assisted thyroidectomy: a prospective, randomized case-control evaluation. Surg endoscopy. (2009) 23:996–1003. doi: 10.1007/s00464-008-0098-3 18806939

[42] Zhang GL Zhang GL Lin YM Li B Gao J Chen YJ. Endoscopic thyroidectomy versus traditional open thyroidectomy for identification of the external branch of the superior laryngeal nerve. Surg Endosc. (2021) 35:2831–7. doi: 10.1007/s00464-020-07718-x 32754826

[43] Huang S Garstka ME Murcy MA Bamford JA Kang SW Randolph GW. Somatosensory evoked potential: Preventing brachial plexus injury in transaxillary robotic surgery. Laryngoscope. (2019) 129:2663–8. doi: 10.1002/lary.v129.11 30671961

[44] Lin P-L Zheng F Shin M Liu X Oh D D’Attilio D. CUSUM learning curves: what they can and can’t tell us. Surg Endoscopy. (2023) 37:7991–9. doi: 10.1007/s00464-023-10252-1 PMC10520215 37460815

