# Supplementary material for: Patient satisfaction and operator proficiency in gasless transaxillary endoscopic thyroidectomy under IONM: a retrospective cohort study
Source: Front Endocrinol (Lausanne). 2024 Oct 9;15:1457571. doi: 10.3389/fendo.2024.1457571 (PMC11496098; doi:10.3389/fendo.2024.1457571)
Supplement: Supplementary file 1 [file DataSheet1.docx]

**University of Washington Quality of Life questionnaire**

**1. Pain**

I have no pain at all

I have mild pain that does not require medication

I have moderate pain that requires regular medication (e.g., ibuprofen)

I have severe pain that must be controlled with narcotics (e.g., morphine) I have very severe pain that cannot be controlled with narcotics (e.g., morphine)

**2. Appearance**

My appearance has not changed

My appearance has changed very slightly

My change in appearance bothers me, but I am able to stay active

I feel severely disfigured and this limits my vitality

I cannot get along with people because of my appearance

**3. Activity**

I am as active as I used to be

I sometimes can't move as fast as I used to, but not very often

I am often tired and although I do go out, I slow down the pace of my life

I don't go out because I don't have the strength

I am often bedridden or in a chair and cannot leave the house

**4. Recreation**

There are no indoor or outdoor restrictions on my recreation

There are things I can't do, but I can still get out and enjoy myself

I have wished many times to go out more, but I cannot

I am severely limited in what I can do and I stay home most of the time to watch TV

I can't enjoy life at all

**5. Swallowing**

I can swallow food like I used to

I can only eat fluids

I can't swallow certain solid foods

I can't swallow because I choke when I swallow food

**6. Chewing**

I can chew food like I used to

I can eat softer foods, but I can't bite into certain foods (e.g., brittle bones).

I can't even eat soft foods

**7. Speech**

I can speak the same as before.

I find it hard to say certain words, but I can be heard clearly on the telephone.

I can't be understood at all.

Only my family and friends can understand me.

**8. Neck and Shoulder**

I have no problems with my neck and shoulder.

My neck and shoulder are stiff and constricted, but they do not interfere with my movement.

My neck and shoulder hurt or I can only bend forward so I have to change my job.

I cannot work because of my neck and shoulder problem.

**9. Taste**

I can taste food normally.

I can only taste some foods.

I can taste most foods normally.

I can't taste food at all.

**10. Saliva**

My saliva is normal.

I have too little saliva.

I have slightly less saliva, but it is sufficient.

I have no saliva at all.

**11. Mood**

My mood is good and not affected by the cancer.

I have some depression because of the cancer.

My mood is generally good, occasionally affected by cancer.

I am extremely depressed because of cancer.

**12. Anxiety**

I am not anxious about my cancer.

I am anxious about my cancer.

I am a little anxious about my cancer.

I am extremely anxious about my cancer.

**The 3 items that affected you the most after surgery?**

Pain

Appearance

Vitality

Recreation

Swallowing

Chewing

Speech

Neck and Shoulder

Taste

Saliva

Emotions

Anxiety

**Synthesized questions**

1. How would you rate the quality of your health-related survival now compared to 1 month before you had cancer?

Better than before

Much better than before

Somewhat worse than before

Much worse than before

2. Overall, how would you rate your health-related quality of survival after surgery?

Excellent

Fairly good

Fair

Poor

Very poor

3. Overall quality of survival includes not only physical health and mental health, but also some other factors such as family, friends, spirituality, personal recreational activities, and a number of other factors that are important to your enjoyment of life, Consider all the things that have an impact on your personal life and evaluate your overall quality of survival after surgery:.

Excellent

Fairly good

Fair

Poor

Very poor

**VOICE HANDICAP INDEX-30**

To assess how much vocal degradation affects your life, please circle the number that corresponds to your situation: 0=None 1=Rarely 2=Sometimes 3=Often 4=Always

| **Part I. FUNCTIONAL** | | | | | | |
| --- | --- | --- | --- | --- | --- | --- |
| F1 | Due to my voice problem people have difficulty hearing my voice | 0 | 1 | 2 | 3 | 4 |
| F2 | It's hard for people to understand me in a noisy environment. | 0 | 1 | 2 | 3 | 4 |
| F3 | When I call my family from across the room, they have trouble hearing me. | 0 | 1 | 2 | 3 | 4 |
| F4 | I will make fewer phone calls. | 0 | 1 | 2 | 3 | 4 |
| F5 | I deliberately avoid talking to people in crowded places. | 0 | 1 | 2 | 3 | 4 |
| F6 | I talk less to friends, neighbors or relatives | 0 | 1 | 2 | 3 | 4 |
| F7 | When talking face to face, people ask me to repeat what I have said | 0 | 1 | 2 | 3 | 4 |
| F8 | It limits my social life. | 0 | 1 | 2 | 3 | 4 |
| F9 | I feel unable to keep up in conversation. | 0 | 1 | 2 | 3 | 4 |
| F10 | My income has been affected. | 0 | 1 | 2 | 3 | 4 |
| **Part II. PHYSICAL** | | | | | | |
| P1 | I feel short of breath when I speak. | 0 | 1 | 2 | 3 | 4 |
| P2 | My voice sounds unstable and changes throughout the day. | 0 | 1 | 2 | 3 | 4 |
| P3 | People would ask me, ‘What's wrong with your voice?’ | 0 | 1 | 2 | 3 | 4 |
| P4 | My voice sounds hoarse. | 0 | 1 | 2 | 3 | 4 |
| P5 | I feel as if I have to work to make a sound. | 0 | 1 | 2 | 3 | 4 |
| P6 | The clarity of my voice is variable. | 0 | 1 | 2 | 3 | 4 |
| P7 | I would try to change my voice to sound different. | 0 | 1 | 2 | 3 | 4 |
| P8 | I struggle to speak. | 0 | 1 | 2 | 3 | 4 |
| P9 | My voice is worse at night. | 0 | 1 | 2 | 3 | 4 |
| P10 | I lose my voice when I speak. | 0 | 1 | 2 | 3 | 4 |
| **Part 3 EMOTIONAL** | | | | | | |
| E1 | My voice makes me nervous when talking to others. | 0 | 1 | 2 | 3 | 4 |
| E2 | Other people find it difficult to hear my voice. | 0 | 1 | 2 | 3 | 4 |
| E3 | I find that others don't understand my voice problems. | 0 | 1 | 2 | 3 | 4 |
| Due to voice problems: | | | | | | |
| E4 | I feel distressed. | 0 | 1 | 2 | 3 | 4 |
| E5 | I have become less outgoing than before. | 0 | 1 | 2 | 3 | 4 |
| E6 | I feel physically defective. | 0 | 1 | 2 | 3 | 4 |
| E7 | I feel annoyed when people ask me to repeat what I have just said. | 0 | 1 | 2 | 3 | 4 |
| E8 | I feel embarrassed when people ask me to repeat what I have just said. | 0 | 1 | 2 | 3 | 4 |
| E9 | I feel inadequate (useless). | 0 | 1 | 2 | 3 | 4 |
| E10 | I feel ashamed. | 0 | 1 | 2 | 3 | 4 |

**The patient scale portion of POSAS**

| PSAS | Score | Description |
| --- | --- | --- |
| Is the scar painful? | 1  ↓  10 | No, no complaints  Yes, worst imaginable |
| Is the scar itching? | 1  ↓  10 | No, no complaints  Yes, worst imaginable |
| Is the color of the scar different? | 1  ↓  10 | Yes, very different  No, as normal skin |
| Is the scar more stif? | 1  ↓  10 | Yes, very different  No, as normal skin |
| Is the thickness of the scar different? | 1  ↓  10 | Yes, very different  No, as normal skin |
| Is the scar irregular? | 1  ↓  10 | Yes, very different  No, as normal skin |
